# Anterior Mediastinal Mass in a Young Marijuana Smoker: A Rare Case of Small-Cell Lung Cancer

**DOI:** 10.1155/2012/754231

**Published:** 2012-04-01

**Authors:** Jiten P. Kothadia, Saurabh Chhabra, Alan Marcus, Michael May, Biren Saraiya, Salma K. Jabbour

**Affiliations:** ^1^Department of Radiation Oncology, The Cancer Institute of New Jersey, Robert Wood Johnson Medical School, University of Medicine and Dentistry of New Jersey, New Brunswick, NJ 08901, USA; ^2^Division of Medical Oncology, Department of Medicine, The Cancer Institute of New Jersey, Robert Wood Johnson Medical School, University of Medicine and Dentistry of New Jersey, New Brunswick, NJ 08901, USA; ^3^Department of Pathology, Robert Wood Johnson Medical School, University of Medicine and Dentistry of New Jersey, New Brunswick, NJ 08901, USA

## Abstract

The use of cannabis is embedded within many societies, mostly used by the young and widely perceived to be safe. Increasing concern regarding the potential for cannabis to cause mental health effects has dominated cannabis research, and the potential adverse respiratory effects have received relatively little attention. We report a rare case of 22-year-old man who presented with bilateral neck lymphadenopathy, fatigue, and sore throat without significant medical or family history. The patient had smoked one marijuana joint three times a week for three years but no cigarettes. Chest CT demonstrated a large anterior mediastinal mass compressing the superior vena cava and mediastinal lymphadenopathy. A final diagnosis of small-cell lung cancer was reached. Although rare, a small-cell lung cancer in this patient should alert the physician that cannabis smoking may be a risk factor for lung cancer.

## 1. Introduction

Cannabis is the most widely consumed illicit drug worldwide, and the relationship between cannabis smoking and lung cancer is suggestive, albeit inconclusive [1]. Cannabis and tobacco smoke contain a similar mix of irritant and toxic chemicals, so it is suspected that cannabis and tobacco have similar side effects [2]. However, the pulmonary effects of smoking cannabis have not been extensively researched, and the few findings are contradictory [2]. We describe a case of small-cell lung cancer in a 22-year-old Caucasian man who had a history of heavy short-term cannabis use.

## 2. Case Presentation

A 22-year-old man presented with bilateral neck lymphadenopathy, fatigue, and sore throat. He was treated for presumed upper respiratory tract infection and subsequent mononucleosis with antibiotics and a short course of steroids, respectively, with no improvement of his symptoms. Approximately one month prior to admission, he noted persistent cough, exertional dyspnea, intermittent right-sided chest pain, hoarseness, solid food dysphagia, and facial and neck swelling. He had no significant medical or family history. The patient had smoked one marijuana joint three times a week for three years but no cigarettes. Examination revealed tachycardia with 130 beats/minute, facial plethora, enlarged nontender cervical neck lymphadenopathy, and decreased right upper lung breath sounds, suggestive of superior vena cava (SVC) obstruction.

**Figure 1:**
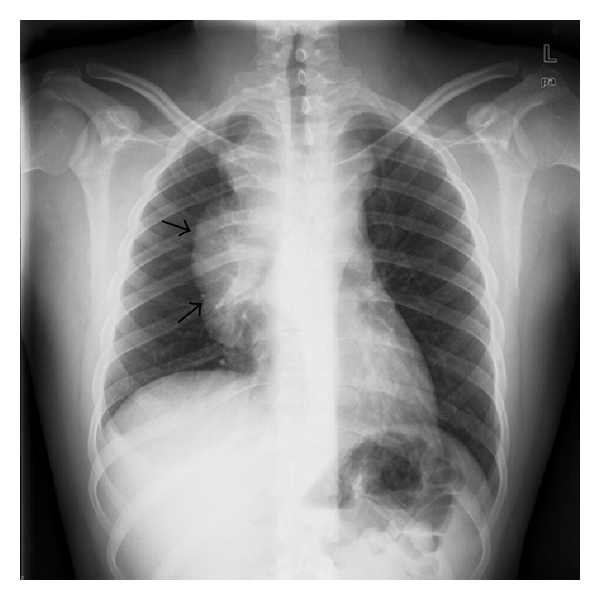
Chest radiograph at initial presentation showing large right hilar mass with right paratracheal soft tissue mass (see arrows).

**Figure 2:**
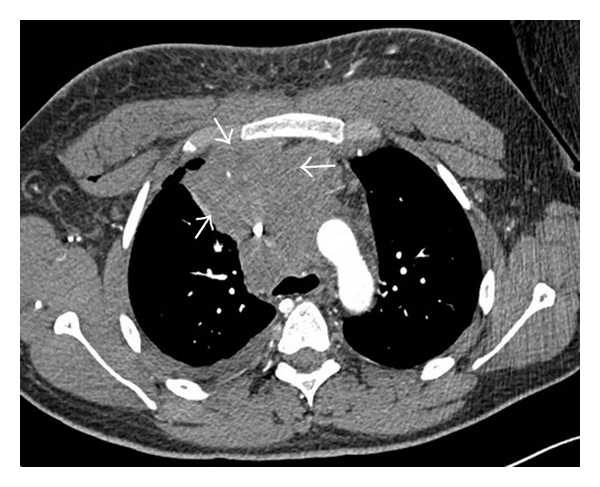
Pretreatment computed tomography of the thorax demonstrating a large anterior mediastinal mass (see arrows).

**Figure 3:**
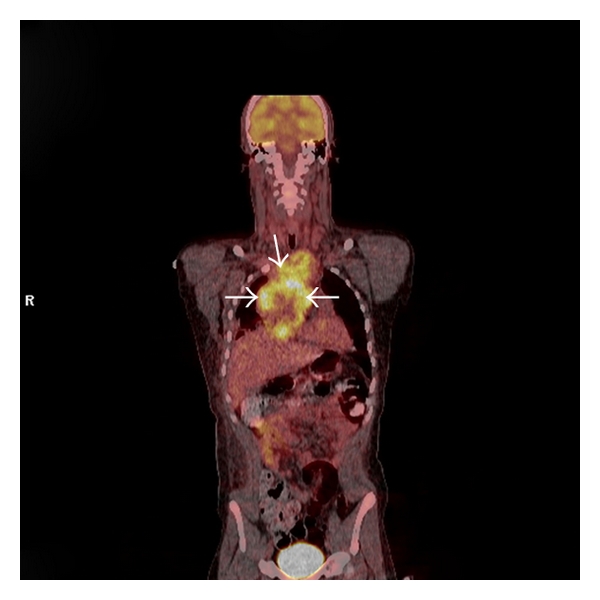
Positron emission tomography (PET) scan showing a hypermetabolic mediastinal mass (see arrows).

Initial chest radiograph (Figure 1) showed a large right hilar mass with right paratracheal soft tissue mass. Chest CT (Figure 2) demonstrated a large anterior mediastinal mass (13 cm × 6 cm) compressing the SVC, right pleural effusion, and subcarinal and pericardiac lymphadenopathy. Positron emission tomography (PET) scan confirmed a hypermetabolic mediastinal mass extending to the subcarinal region and inferiorly to diaphragmatic surface along the right side of the heart with maximum standardized uptake value of 6.5 (Figure 3). The differential diagnosis included lymphoma, germ cell tumor, and lung cancer. Laboratory studies showed nonreactivity for HIV, negative hepatitis B and C serology, undetectable *β*-HCG, and normal levels of *α*-fetoprotein, and lactate dehydrogenase. Testicular ultrasound was normal.

**Figure 4:**
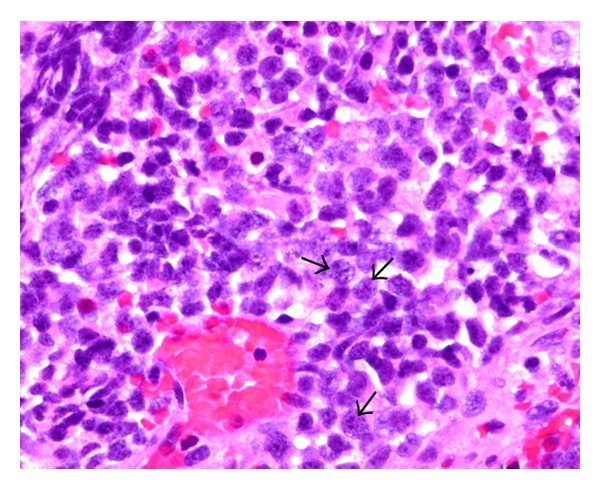
The tumor cells (see arrows) exhibit features of small-cell carcinoma: nuclear molding, high nuclear-to-cytoplasm ratio, and fine chromatin pattern/“salt and pepper” nuclei. H and E (hematoxylin and eosin) staining.

**Figure 5:**
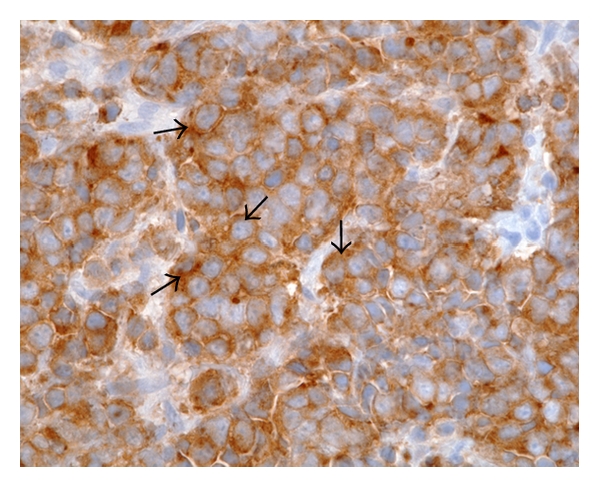
Positive synaptophysin stain, consistent with neuroendocrine origin (see arrows).

Core biopsy of the mediastinal mass under CT guidance contained little viable tissue due to extensive necrosis. Nevertheless, immunohistochemistry (IHC) showed negativity for lymphoid markers (CD3, TdT, CD20, PAX-5, and CD10) and germ cell markers (AFP, PLAP, CD30, and OCT-4). Bone marrow biopsy was normocellular. Open thoracotomy biopsy of the mediastinal mass revealed small blue cells with pleomorphic nuclei arranged in nests within dense fibroconnective tissue. The tumor cells exhibited features of small-cell carcinoma: nuclear molding, high nuclear-to-cytoplasm ratio, and fine chromatin pattern/“salt and pepper” nuclei (Figure 4). IHC showed strong immunoreactivity with synaptophysin consistent with neuroendocrine origin (Figure 5), CK8/18, CK7, and weak focal immunoreactivity for chromogranin and TTF-1. The immunostains were negative for the following markers: CD99, desmin, SMA, LCA, vimentin, WT-1, *β*-HCG, and CK20. This staining pattern confirmed poorly differentiated, high-grade neuroendocrine carcinoma, or small-cell lung cancer (SCLC).

Staging workup including PET-CT, bone scan, and brain MRI showed no extrathoracic disease, and his disease was considered limited stage SCLC (T4 N3 M0) as the pleural effusion showed no cytological evidence of malignant cells and was PET-negative.

Initiation of urgent radiation therapy with cisplatin and etoposide relieved his symptoms. The patient was free of disease for approximately 16 months and subsequently experienced metastatic disease in the liver and para-aortic lymph nodes.

## 3. Discussion

Marijuana is the most commonly used illicit drug in the USA. In 2009, there were 16.7 million (6.6% of the US population) pastmonth users [3]. The main active chemical in marijuana is delta-9-tetrahydrocannabinol (THC). The use of cannabis is embedded within many societies, mostly used by the young and widely perceived to be safe. Several studies have shown that cannabis smoking may have a greater potential than tobacco smoking to cause lung cancer [4, 5]. Cannabis smoke is qualitatively similar to tobacco smoke; although it contains 50–70% more carcinogenic hydrocarbons than tobacco smoke [6].

Cannabis is usually smoked as a cigarette (joint) or in a pipe. It is also smoked in blunts, which are cigars that have been emptied of tobacco and refilled with a mixture of cannabis and tobacco. This mode of delivery combines marijuana's active ingredients with nicotine and other harmful chemicals. Cannabis cigarettes are less densely packed than tobacco cigarettes and tend to be smoked without filters to a smaller butt size, leading to higher concentrations of smoke inhaled [5, 7]. Furthermore, smokers of cannabis inhale more deeply and hold their breath longer [8], which further increase the lungs' exposure to carcinogenic smoke. These factors are likely to be responsible for the greater absorption of carbon monoxide from cannabis joints, compared with a tobacco cigarette of similar size despite similar carbon monoxide concentrations in the smoke inhaled. Moreover, THC causes modest short-term bronchodilation [8]. Regular marijuana smoking produces a number of long-term pulmonary consequences, including chronic cough and sputum. It can cause histopathologic evidence of widespread airway inflammation and injury with IHC evidence of dysregulated growth of respiratory epithelial cells that may be precursors to lung cancer [8]. Several experimental studies have demonstrated precancerous histological [9–11] and molecular [12] abnormalities in the respiratory tracts of cannabis smokers.

The carcinogenic effects of cannabis smoke have been demonstrated both *in vitro *and *in vivo* models [13]. Tumors may either directly release factors or orchestrate immune suppressive networks by inducing host immune cell production of inhibitory cytokines. For example, the immune inhibitory cytokines, IL-10 and TGF-*β*, can be produced or induced by tumors, causing limitations in immune reactivity against the tumor [13–15]. Conversely, there is also evidence that THC may have anticarcinogenic effects [16–18]. Epidemiological evidence for an association between cannabis and lung cancer is limited and conflicting. Case control studies published to date have been limited by the inability to quantify use [19], confounding with combined cannabis and tobacco use. Studies undertaken in populations in which use may have serious legal consequences result in potential information bias and poor response rates [5].

At 22 years of age, this patient was quite young to be diagnosed with small-cell lung cancer. In the United States, there have been 0.03 cases per 1000 of lung cancer reported in patients younger than 39 years. In this case, there was a no cigarette smoking exposure and no known genetic predisposition to developing lung cancer. A recent case control study from Aldington et al. [5] of young New Zealand residents found that each joint-year (one joint per day for 1 year) increased the risk of lung cancer by 8%. Therefore, smoking one joint of cannabis per day produces a similar risk of malignancy as smoking 20 cigarettes per day [5]. This case report suggests an association between cannabis smoking and increased risk of lung cancer. Future studies, especially among heavy and long-term smokers of cannabis and among nonsmokers of tobacco would be desirable to further corroborate our results, but such populations may be difficult to find.

## 4. Conclusion

From a public health perspective, physicians and patients should be aware that smoking cannabis may be associated with development of lung cancer. Larger studies and pooled analysis of published data that include subjects who smoked cannabis without tobacco are needed to evaluate the association between use of cannabis and lung cancer.

## References

[1] Berthiller J, Straif K, Boniol M (2008). Cannabis smoking and risk of lung cancer in men: a pooled analysis of three studies in Maghreb. *Journal of Thoracic Oncology*.

[2] Grendelmeier P (2010). Cannabis and the lung—chill or kill?. *Therapeutische Umschau*.

[3] (2010). Summary of the 2009 national survey on drug use and health. *Journal of Pain & Palliative Care Pharmacotherapy*.

[4] Voirin N, Berthiller J, Benhaïm-Luzon V (2006). Risk of lung cancer and past use of cannabis in Tunisia. *Journal of Thoracic Oncology*.

[5] Aldington S, Harwood M, Cox B (2008). Cannabis use and risk of lung cancer: a case-control study. *European Respiratory Journal*.

[6] Rickert WS, Robinson JC, Rogers B (1982). A comparison of tar, carbon monoxide and pH levels in smoke from marihuana and tobacco cigarettes. *Canadian Journal of Public Health*.

[7] Tashkin DP, Gliederer F, Rose J (1991). Tar, CO and Δ9THC delivery from the 1st and 2nd halves of a marijuana cigarette. *Pharmacology Biochemistry and Behavior*.

[8] Tashkin DP (2005). Smoked marijuana as a cause of lung injury. *Monaldi Archives for Chest Disease*.

[9] Hart S, Fischer OM, Ullrich A (2004). Cannabinoids induce cancer cell proliferation via tumor necrosis factor *α*-converting enzyme (TACE/ADAM17)-mediated transactivation of the epidermal growth factor receptor. *Cancer Research*.

[10] Fligiel SEG, Roth MD, Kleerup EC, Barsky SH, Simmons MS, Tashkin DP (1997). Tracheobronchial histopathology in habitual smokers of cocaine, marijuana, and/or tobacco. *Chest*.

[11] Gong H, Fligiel S, Tashkin DP, Barbers RG (1987). Tracheobronchial changes in habitual, heavy smokers of marijuana with and without tobacco. *American Review of Respiratory Disease*.

[12] Barsky SH, Roth MD, Kleerup EC, Simmons M, Tashkin DP (1998). Histopathologic and molecular alterations in bronchial epithelium in habitual smokers of marijuana, cocaine, and/or tobacco. *Journal of the National Cancer Institute*.

[13] Zhu LX, Sharma S, Stolina M (2000). Δ-9-tetrahydrocannabinol inhibits antitumor immunity by a CB2 receptor- mediated, cytokine-dependent pathway. *Journal of Immunology*.

[14] Seo N, Tokura Y, Takigawa M, Egawa K (1999). Depletion of IL-10- and TGF-*β*-producing regulatory *γδ* T cells by administering a daunomycin-conjugated specific monoclonal antibody in early tumor lesions augments the activity of CTLs and NK cells. *Journal of Immunology*.

[15] Huang M, Stolina M, Sharma S (1998). Non-small cell lung cancer cyclooxygenase-2-dependent regulation of cytokine balance in lymphocytes and macrophages: up-regulation of interleukin 10 and down-regulation of interleukin 12 production. *Cancer Research*.

[16] Melamede RJ (2005). Cannabis and tobacco smoke are not equally carcinogenic. *Harm Reduction Journal*.

[17] Ligresti A, Moriello AS, Starowicz K (2006). Antitumor activity of plant cannabinoids with emphasis on the effect of cannabidiol on human breast carcinoma. *Journal of Pharmacology and Experimental Therapeutics*.

[18] Munson AE, Harris LS, Friedman MA (1975). Antineoplastic activity of cannabinoids. *Journal of the National Cancer Institute*.

[19] Sasco AJ, Merrill RM, Dari I (2002). A case-control study of lung cancer in Casablanca, Morocco. *Cancer Causes and Control*.

